# Nano Iron Versus Bulk Iron Forms as Functional Feed Additives: Growth, Body Indices, Hematological Assay, Plasma Metabolites, Immune, Anti-oxidative Ability, and Intestinal Morphometric Measurements of Nile tilapia, *Oreochromis niloticus*

**DOI:** 10.1007/s12011-023-03708-x

**Published:** 2023-06-23

**Authors:** Eman Y. Mohammady, Mohamed A. Elashry, Mohamed S. Ibrahim, Mohamed Elarian, Shimaa M. R. Salem, Ehab R. El-Haroun, Mohamed S. Hassaan

**Affiliations:** 1 National Institute of Oceanography and Fisheries, NIOF, Cairo, Egypt; 2 Department of Animal Production, Fish Research Laboratory, Faculty of Agriculture at Moshtohor, Benha, University, Tanta, 13736 Qalubia Egypt; 3 Central Laboratory for Aquaculture Research, Abou-Hammad, AbbassaSharkia, Egypt; 4 Faculty of Veterinary Medicine, Department of Animal Nutrition and Nutritional Deficiency Diseases, Mansoura University, Mansoura, Egypt; 5 Animal Production Department, Faculty of Agriculture, Cairo University, Cairo, Egypt

**Keywords:** Feed additive, Bulk-Fe_2_O_3_, Nano-Fe_2_O_3_, Growth indices, Serum chemistry, Gut metrics, *Oreochromis niloticus*

## Abstract

The current study aimed to compare the utilization efficiency of iron (Fe) feed additives from either bulk or nano sources in Nile tilapia, *Oreochromis niloticus* diets on growth, haematological, immunity, anti-oxidative, and intestinal topography capacities. Five isonitrogenous and isoenergetic diets were performed; the basal diet served as a control with no Fe added, whereas the experimental diets were shaped by adding bulk-Fe_2_O_3_ and nano-Fe_2_O_3_ to the basal diet to preserve Fe levels at 0.2 and 0.4 mg kg^−1^, respectively. Results indicated that superior growth performance was recorded in fish-fed diets supplemented with 0.4 nano-Fe_2_O_3_ mg kg^−1^ diet. In addition, the highest (*P* ≤ 0.05) survival rate, absorption area of villous (AAV), mucosal to serosal amplification ratio (MSR), and villi parameters (height and width) were noticed in fish fed diet enrichment with either bulk or nano-Fe_2_O_3_ source. However, the superiority observed in nano-Fe_2_O_3_ fish groups. Also, the highest values of plasma albumin, total protein, high-density lipoprotein cholesterol (HDL-C), white blood cells (WBCs), and lymphocyte absolute count (LYM) (*P* ≤ 0.05) recorded in fish fed a diet supplemented with nano-Fe_2_O_3_ versus the basal diet. Moreover, the highest values of catalase (CAT), glutathione peroxidase (GPx), superoxide dismutase (SOD), and plasma lysozyme activity (*P* ≤ 0.05) were observed in fish fed 0.4 mg/kg^−1^ nano-Fe_2_O_3_, while the lowest value was recorded in fish fed the control diet. The best value of malondialdehyde activity (*P* ≤ 0.05) recorded in a fish-fed diet supplemented with 0.4 mg/kg^−1^ nano-Fe_2_O_3._ The current findings emphasize the importance of including Fe to improve fish growth, immunity, antioxidant capabilities, and intestinal structure, primarily with a nano-Fe source, which demonstrated a more effective function in satisfying Nile tilapia dietary Fe requirements and improving the aforementioned parameters.

## Introduction

Nowadays, achieving food security through seafood production forces the farmers and aquaculture stakeholders towards intensification of tilapia’s production technique to secure the global demand for food fish with a global production of 6.5 million MT in 2018 [1, 2]. The sustainability of aquaculture production relies on many factors, such as the provision of balanced, complete diets that supply essential nutrients qualitatively and quantitatively to ensure maximum fish growth, control disease outbreaks and pathogen infections, and maintain health status at a high standard level [1–5]. Iron is one of the essential micro-minerals and can maintain some physiological processes, including oxygen transport, fat oxidation, haemoglobin formation, stress tolerance, optimal enzyme activity, DNA synthesis, ATP production, electron transport, and improved immunological function [6, 7]. Iron deficiency has been associated with microcytic anaemia, growth depression, changes in intestinal morphology (villus width/length), immune suppression, haematological abnormalities, and disease susceptibility [8]. Fish can obtain the required iron either from the surrounding water via the gills or from their diet. However, the dietary source is considered the main route due to the limited absorption through the gills. As well as using dietary feed supplements of high availability are considered critical points should be followed during aquafeed formulation, to meet the nutritional demand, improve feed quality, and maximize the productivity of farmed fish [9, 10].

The evolution of nanotechnology science has resulted in different kinds of nanoparticles (NPs) that are necessarily used in biomedical sciences, agriculture, and industry [11]. Any particle having at least one dimension less than 100 nm is referred to as a nanoparticle, and as a result, its properties differ from those of their bulk materials [12]. In aquaculture, nanoparticles can reduce the prevalence and spread of pathogenic pathogens. [13] stated that some metal nanoparticles are capable of inhibiting the growth of several bacterial and fungal species, including freshwater cyanobacteria.

Iron supplements are available in different forms: organic, inorganic, and nanoparticles, varying in the degree of their bioavailability. It has been reported that nanoparticles have a higher absorption and utilization rate compared to other chemical forms, due to it has energy level, small size, active atoms content, and ability to pass cell membranes faster [14]. These causes explained the findings demonstrated previously by [8] in Rohu *Labeo rohita* fed iron nanoparticles. Thus, antioxidants, such as iron, have been added to farm animal feed to avoid deteriorative oxidative processes or to promote oxidative stability in order to maintain food quality and nutritional value [15].

Therefore, the present study investigated the efficacy of iron supplements from different sources (bulk-Fe_2_O_3_ and nano-Fe_2_O_3_) on performance, fillet nutrient compositions, haematological parameters, the blood biochemical profile, antioxidant enzymes, immune function, and the histomorphometry of the intestine of Nile tilapia.

## Material and Methods

### Experimental Diet Preparation

The basal diet (Diet 1; Table 1) was formulated to fulfil the requirements of Nile tilapia according to the [1]. Two sources of iron (Fe), the bulk-Fe_2_O_3_ and nano-Fe_2_O_3_, were added to the control diet at 0.2 (Diet 2) and 0.4 (Diet 3) mg kg^−1^. Also, nano-Fe_2_O_3_ was added to the control diet at 0.2 (Diet 4) and 0.4 (Diet 5) mg /kg^−1^ diet. Fe was supplemented from bulk (Fe_2_O_3_) and nanoscale sources. Nano-Fe_2_O_3_ (Sigma-Aldrich, 207,780–500: 99%, USA) was used as the Fe source. The sizes of the elemental nano-Fe particles are less than 73 μm. Bulk-Fe_2_O_3_ and nano-Fe_2_O_3_ were thoroughly mixed with the control diet; after that, 300 ml of water per kg of diet was added to form a dough. The feed mixture was pelleted (2-mm diameter die) via a laboratory pelletizer (CPM, California Pellet Mill Co., San Francisco, California, USA). The feed pellets were kept chilled at 4 °C until use. The proximate composition of the experimental diets nutrients was analyzed following [16] procedures. The dietary Fe contents were 124.72 (control), 124.92 (Diet 2), 125.12 (Diet 3), 124.92 (Diet 4), and 125.12 (Diet 5) mg kg^−1^ diet.

**Table 1:** Ingredients and proximate nutrient composition percentage of the control diet

Ingredients	%
Fish meal (65%)	10
Soybean meal (45%)	40.8
Corn gluten meal	6.00
Yellow corn	19.5
Wheat flour	18.5
Soybean oil	3
Vitamin mixture^*^	0.8
Mineral mixture^**^	0.5
DiCaP	0.6
Choline chloride	0.2
Stay C^***^	0.1
Proximate analysis	%
Crude protein	31.84
Crude lipid	5.44
Ash	4.923
Crude fiber	3.827
DE (Kcal/kg)	3005
Ca	0.784
P	0.810
Fe (mg/kg)	124.72

^*^Vitamin (g/kg premix): thiamin HCl, 0.44; riboflavin, 0.63; pyridoxine HCl, 0.91; DL pantothenic acid, 1.72; nicotinic acid, 4.58; biotin, 0.21; folic acid, 0.55; inositol, 21.05; menadione sodium bisulfite, 0.89; vitamin A acetate, 0.68; vitamin D3, 0.12; dL-alpha-tocoperol acetate, 12.63; alpha-cellulose, 955.59

^**^Trace mineral, iron-free (g/100 g premix): cobalt chloride, 0.004; cupric sulfate pentahydrate, 0.25; magnesium sulfate anhydrous, 13.862; manganous sulfate monohydrate, 0.650; potassium iodide, 0.067; sodium selenite, 0.010; zinc sulfate hepahydrate, 13.193; alpha-cellulose, 67.964

^***^Stay C®, (L-ascorbyl-2-polyphosphate 35%). Abbreviations: *DE*, digestible energy (Kcal/kg); *Ca*, calcium; *P*, phosphorous; *Fe*, iron

### Experimental Protocol and Animal Care

Mono-sex Nile tilapia fingerlings were purchased from the private farm of Kafr El-Shaikh and adapted in central laboratory for Aquaculture Research, Abbassa, Abou-Hammad, Sharkia, Egypt, in two circular fiberglass tanks (1 m^3^ for each) for two weeks and fed the control diet containing 31.84% crude protein at a rate of 5% of the whole biomass three times daily before the start of the experiment. After adaptation, fish were fasted for 24 h. Four hundred and fifty healthy tilapias (9.10 ± 0.014 g) were randomly allocated to 15 tanks (80 × 50 × 50 cm; 200 L for each), representing the five groups with three replicates, 30 fish per aquarium. Fish fed at 3% of their body weight the experimental diets three times/day by hand for 12 weeks. Fish were weighed once every two weeks and the daily ration was attuned rendering to weight gain. During the experiment, water temperature was recorded daily with a mercury thermometer suspended at 15-cm depth. pH was determined by using a pH meter (Orion pH meter, Abilene, Texas, USA), while dissolved oxygen (mg/L) was measured using YSI model 56 oxygen meter (YSI Company, Yellow Springs Instrument, Yellow Springs, Ohio, USA). Total ammonia was measured using DREL/2 HACH kits (HACH Co., Loveland, Co. USA). During the feeding trial, the water quality parameters averaged (± standard deviation): Water temperature 26.8 ± 0.3 °C; dissolved oxygen 5.98 ± 0.22; pH values 8.17 ± 0.35; total ammonia 0.16 ± 0.01 mg/L. All tested water quality criteria (temperature, dissolved oxygen, pH value, and total ammonia) were within the acceptable limits for rearing Nile tilapia.

### Growth and Body Indices Estimation

Initial body weight (g) (IBW) and final body weight (g) (FBW) of individual fish were recorded for all fish/each tank at the initiation and the termination of the experiment. Also, the number of fish in each tank was counted and recorded. Weight gain (WG) was calculated as follows: WG = FBW (g) − IBW (g); condition factor (K) was calculated using the following formula: *K* = (*W*/L3) × 100, where *W* = weight of fish in grams and *L* = total length of fish in cm; specific growth rate (SGR) = 100 × (Ln W2 − Ln W1)/*T*, where Ln = natural log, W1 = initial body weight, W2 = final body weight, and *T* = study period (84 days); feed conversion ratio (FCR) was calculated according to the equation: FCR = feed intake (g)/weight gain (g); protein efficiency ratio (PER) = weight gain (g)/protein ingested (g); survival rate percentage (SR) = 100 × (total number of fish at the end of the experiment / total number of fish at the start of the experiment) as described previously in [17] and renowned in the footnote of Table 2.

**Table 2:** Growth performance and feed utilization of Nile tilapia as affected by dietary additives of bulk and nano-Fe_2_O_3_

	Experimental treatments					*P* value
	Control	Bulk-Fe_2_O_3_ (mg kg^−1^ diet)		Nano-Fe_2_O_3_ (mg kg^−1^ diet)		
		0.2	0.4	0.2	0.4	
Initial body weight (IBW; g fish^−1^)	9.09 ± 0.014	9.08 ± 0.014	9.10 ± 0.014	9.11 ± 0.014	9.12 ± 0.014	0.2903
Final body weight (FBW; g fish^−1^)	41.20 ± 0.77^d^	50.16 ± 0.717^c^	51.13 ± 0.704^c^	57.87 ± 691^b^	60.19 ± 0691^a^	0.0001
Final body length (FBL)	13.03 ± 0.033^e^	14.30 ± 0.035^d^	14.57 ± 0.034^c^	14.85 ± 0.036^b^	15.08 ± 0.036^a^	0.0001
Condition factor (K)	1.86 ± 0.015^a^	1.72 ± 0.014^c^	1.65 ± 0.013^d^	1.77 ± 0.013^b^	1.76 ± 0.013b^c^	0.0001
Weight gain (WG; g fish^−1^)	32.09 ± 0.015^d^	40.41 ± 0.015^c^	41.63 ± 0.015^c^	48.45 ± 0.015^b^	51.04 ± 0.015^a^	0.0001
Specific growth rate (SGR; % day^−1^)	1.78 ± 0.015^d^	2.01 ± 0.015^c^	2.03 ± 0.015^c^	2.18 ± 0.015^b^	2.24 ± 0.015^a^	0.0001
Fish survival (FS %)	80 ± 0.015^b^	93.33 ± 0.015^a^	96.67 ± 0.015^a^	100 ± 0.015^a^	100 ± 0.015^a^	0.0017
Feed intake (FI; g fish^−1^)	59.69 ± 0.015^c^	70.72^b^	70.90 ± 0.015^b^	78.50 ± 0.015^a^	78.43 ± 0.015^a^	0.0001
Feed conversion ratio (FCR)	1.86 ± 0.015^a^	1.75 ± 0.015^b^	1.70 ± 0.015^c^	1.62 ± 0.015^d^	1.54 ± 0.015^e^	0.0001
Protein efficiency ratio (PER)	1.79 ± 0.015^d^	1.90 ± 0.015^c^	1.96 ± 0.015^c^	2.04 ± 0.015^b^	2.12 ± 0.015^a^	0.0001

Means followed by different small letters in the same row are significantly different (*P* < 0.05, one-way ANOVA)

WG = final weight (g) − initial weight (g); specific growth rate (SGR) = LnW2 − LnW1/t (days), where Ln = the natural log; W1 = initial fish weight, W2 = the final fish weight in grams and *t* = period in days; FCR = feed intake (g)/weight gain (g); protein efficiency ratio (PER) = weight gain (g)/protein ingested (g); Survival rate percentage (SR) = 100 × (total number of fish at the end of the experiment / total number of fish at the start of the experiment)

### Sample Collection

At the end of the growth period, fish were deprived for 24 h and then anesthetized with tricaine methanesulfonate (MS222) at 150 mg/L [18]. Then, total number and weight of fish in each tank were recorded to calculate the final body weight, weight gain, and survival. Blood samples (500 micron) were taken from the caudal vein of three fish per each replicate by using 10% ethylenediaminetetraacetate (EDTA), then separated into two groups. The first blood group was separated to test the hematological parameters. While the second group of blood was centrifuged at 3000 g for 10 min, to get the blood plasma. The obtained plasma samples were saved at − 20 °C for further analysis. After blood collection, individual fish weight and length were recorded for later estimation of condition factor. Then, fish dissected, samples from anterior and posterior intestine were separated for histomorphometry determination. Intestinal samples were fixed in 10% neutral-buffered formalin until examination [19]. Additionally, other three fish from each replicate were anaesthetized by MS222 at 150 mg/L, homogenized, dried, and stored at − 20 °C for subsequent fish flesh proximate and Fe content analysis.

### Sample Analysis

#### Blood Assay

Hematocrit (%) was analyzed according to [20] procedures. Hemoglobin (Hb, g dL^−1^) was determined using hemoglobin kits (cat. no. KT-731), which is a standardized procedure of the cyanmethemoglobin method. The RBCs (× 10^12^ L^−1^) and WBCs (× 10^9^ L^−1^) numbers were counted by the indirect method described by [21]. The differential counting of WBC was determined according to [22] method. Mean corpuscular volume (MCV, fl), mean corpuscular hemoglobin (pg) (MCH), and mean corpuscular hemoglobin concentration (MCHC) (g dL^−1^) were measured using methodology reported by [23]. Oxygen carrying capacity was calculated by multiplying the Hb content with 1.25 oxygen combining power of Hb g^−1^ [24].

Total protein and albumin (g dL^−1^) of blood plasma were analyzed following [25, 26]. While globulin (g dL^−1^) was calculated by withdrawing albumin from total protein according to [27]. The liver enzyme activity, aspartate aminotransferase (AST), and alanine aminotransferase (ALT) (U L^−1^) were determined as stated by [20]. Plasma total cholesterol (mg dL^−1^), triglyceride (mg dL^−1^), high-density lipoprotein cholesterol (HDL-C), and low-density lipoprotein cholesterol (LDL-C) (mg dL^−1^) were estimated according to [28].

### Plasma Lysozyme Activity and Antioxidant Biomarkers

Plasma lysozyme activity was measured by using the turbidimetric approach according to [29]. The oxidative enzymes including catalase (CAT), superoxidase dismutase (SOD), glutathione peroxidase (GPx), and melanodialdehyde (MDA) activities were measured using according to [30]. The liver of three fish from each replicate were weighed, rinsed and grinded in glass homogenizer tubes with ice-cold saline (to 0.1 g of liver was added 0.9 mL saline, pH 7.0), and centrifuged at 3000 g for 10 min. The supernatant was collected for assays of SOD. SOD was measured using water-soluble tetrazolium salt as a superoxide detector and expressed as units per milligram protein. For CAT activity assay, a mixture of 2.5 ml of phosphate buffer (pH 7.0), 2 ml of H_2_O_2_ solution, and 0.5 ml of sample was added to each tube. The hydrogen peroxide (H_2_O_2_ 30 mM) was used as a substrate and the decrease in H_2_O_2_ concentration at 22 °C was measured spectrophotometrically at 240 nm for 1 min and expressed as specific activities (U/g protein). MDA activity was determined according to [31]. GPx level was measured using diagnostic kits (Bio-diagnostics, Giza, Egypt) following the manufacturer’s instructions according to the method of [32].

### Histomorphometry Examination of the Intestine

The histomorphometry parameters were determined by using a Rotatory Microtome (Reichert Technologies); the longitudinal and transverse slices, each 6 m thick, were cut and stained with haematoxylin and eosin in accordance with usual protocol. The light microscope supplied with a full HD microscopic camera and image processing software Olympus digital camera (Olympus LC20) was fixed on an Olympus microscope (Olympus BX-50) with a 1/2 × image adapter, and a × 40 objective was used to examine the tissue sections. Image analysis software was used to calculate the mean villus height (measured from the base to the top) for statistical analysis. The area of the absorption surface was determined as described by [32].

### Fish Flesh Nutrient Composition

The proximate chemical analysis of fish fillet was analyzed following the technique revealed by [16]. Dry matter was measured after drying the samples in an oven (105 °C) for 24 h. Crude protein was analyzed by micro-Kjeldah method, N% × 6.25 (using Kjeltech auto analyzer, Model 1030; Tecator) and crude fat by Soxhlet extraction with diethyl ether (40–60° C). Ash was determined by ignition at 550 °C for 12 h. Fe concentrations in fish fillet were determined using atomic emission spectrophotometer (IRIS Advantage; Thermo Jarrell Ash Corporation) using standard Fe concentrations [16].

### Statistical Analysis

Data generated are presented as mean ± standard error (SE), and were analyzed by using Statistical Analysis System**,** SAS [33] to identify the significant difference across various treatments, one-way analysis of variance (one-way ANOVA), and the [34] new multiple range test were applied. The data were found significant at *P* ≤ 0.05.

## Results

### Growth and Body Traits

Tilapia’s growth (FBW, WG, and SGR), feed utilization (FI and PER), and body survival rate markedly increased (*P* ≤ 0.05) in fish received diet supplied with Fe compared to basal diet (Table 2). The best (*P* ≤ 0.05) fish performance observed in tilapia group fed on 0.4 nano-Fe_2_O_3_ mg kg^−1^ diet.

### Intestinal Morphometry

The morphometric indices of the intestine structures are illustrated in Table 3. Villi height and width measures in anterior and posterior intestine significantly (*P* < 0.05) increased by dietary supplementation of Fe, the highest significant (*P* < 0.05) measures recorded in nano-Fe_2_O_3_ fish groups. Absorption area of villous (AAV) of anterior intestine noticeably increased significant (*P* < 0.05) in fish fed 0.2 mg/kg^−1^ nano-Fe_2_O_3_, and in posterior intestine AAV and MSR values improved in fish fed nano-Fe_2_O_3_ at both levels 0.2 and 0.4 mg/kg^−1^. Mucosal to serosal amplification ratio (MSR) of anterior intestine significant (*P* < 0.05) rose in fish group received 0.4 mg/kg^−1^ nano-Fe_2_O_3_.

**Table 3:** Histomorphometric of intestine of Nile tilapia as affected by dietary additives of bulk and nano-Fe_2_O_3_

	Experimental treatments					*P* value
	Control	Bulk-Fe_2_O_3_ (mg kg^−1^ diet)		Nano-Fe_2_O_3_ (mg kg^−1^ diet)		
		0.2	0.4	0.2	0.4	
*Anterior intestine*						
Villi height	412.75 ± 6.31^c^	469.5 ± 2.39^b^	479 ± 5.99^b^	675.8 ± 6.78^a^	689.5 ± 6.74^a^	0.001
Villi width	27 ± 0.56^c^	31 ± 0.62^b^	30 ± 0.38^b^	28.5 ± 0.98^b^	39.5 ± 0.88^a^	0.008
Goblet cell number	25.10 ± 0.47^e^	31.00 ± 0.65^d^	37.00 ± 0.65^c^	41.10 ± ^ab^	48.52 ± ^a^	0.001
MSR^‡‡^	7.27 ± 0.32^c^	7.70 ± 0.65^c^	7.17 ± 0.24^c^	10.09 ± 0.87^b^	13.23 ± 0.25^a^	0.001
AAV^††^	43.76 ± 0.98^b^	44.83 ± 1.01^b^	43.40 ± 0.97^b^	61.09 ± 0.78^a^	46.91 ± 0.67^b^	0.001
Crypt width	59.45 ± 1.22^b^	63.83 ± 1.57^a^	64.82 ± 1.78^a^	60.5 ± 2.33^a^	46.42 ± 2.01^c^	0.021
*Posterior intestine*						
Villi height	189 ± 5.16^d^	198 ± 5.19^c^	258.5 ± 4.10^b^	276 ± 6.17^a^	297 ± 4.37^a^	0.049
Villi width	56 ± 2.22^c^	69 ± 3.21^b^	71.5 ± 3.14^b^	72.5 ± 4.71^b^	87 ± 5.23^a^	0.038
Goblet cell number	41.50 ± 1.28	40.00 ± 1.25	39.00 ± 3.12	42.00 ± 2.14	43.00 ± 1.87	0.554
MSR^‡‡^	3.99 ± 1.12^a^	4.92 ± 1.78^b^	5.52 ± 1.72^b^	7.85 ± 0.98^a^	7.67 ± 1.01^a^	0.01
AAV^††^	9.50 ± 1.11^c^	11.48 ± 0.87^c^	12.02 ± 0.56^c^	13.33 ± 1.01^a^	16.20 ± 0.98^a^	0.782
Crypt width	40.93 ± 1.01^b^	43.84 ± 1.05^b^	54.42 ± 0.98^a^	35.93 ± 0.97^c^	30.65 ± 0.99^c^	0.001

Means followed by different small letters in the same row are significantly different (*P* < 0.05, one-way ANOVA). Abbrev: *MSR*, mucosal to serosal amplification ratio; *AAV*, absorption area of villous

### Haematological Analysis

Haematological parameters analysis is stated in Table 4. Hemoglobin concentration was the best (*P* ≤ 0.05) in fish received 0.4 nano-Fe_2_O_3_ mg kg^−1^ diet. Hematocrit percent improved (*P* ≤ 0.05) by Fe supplementation compared to the control free iron diet, whereas the highest (*P* ≤ 0.05) hematocrit percent noticed in those fed diet provided with nano-Fe_2_O_3_. Moreover, WBCs and LYM cell count considerably rose (*P* ≤ 0.05) by dietary addition of Fe compared to the control free iron diet, the best values measured in fish administered 0.2–0.4 mg kg^−1^ Nano-Fe supplemented diets compared to those fed other diets.

**Table 4:** Hematological parameters of Nile tilapia as affected by dietary additives of bulk and nano-Fe_2_O_3_

	Experimental treatments					*P* value
	Control	Bulk-Fe_2_O_3_ (mg kg^−1^ diet)		Nano-Fe_2_O_3_ (mg kg^−1^ diet)		
		0.2	0.4	0.2	0.4	
Hb (g dL^−1^)	11.40 ± 1.13^b^	12.08 ± 1.18^b^	12.47 ± 2.11^b^	12.78 ± 1.13^b^	15.13 ± 1.51^a^	0.02617
Hct (%)	28.26 ± 1.11^c^	32.94 ± 2.02^b^	35.22 ± 2.00^b^	39.98 ± 1.98^a^	38.46 ± 1.97^a^	0.026
RBCs (× 10^12^ L^−1^)	3.16 ± 0.26	3.28 ± 0.35	3.27 ± 0.45	3.37 ± 025	3.29 ± 0.38	0.9842
MCV (fl)	121.86 ± 2.32	122.26 ± 1.39	122.28 ± 1.88	119.75 ± 2.17	117.05 ± 1.85	0.9904
MCH (pg)	36.22 ± 2.30	37.83 ± 1.58	39.05 ± 1.22	38.44 ± 1.74	47.34 ± 1.42	0.4452
MCHC (g dL^−1^)	29.76 ± 1.22	31.05 ± 1.47	31.85 ± 1.21	32.03 ± 1.25	40.31 ± 1.11	0.3717
WBCs (× 10^9^ L^−1^)	196.28 ± 5.32^d^	202.56 ± 7.12^c^	202.44 ± 4.38^c^	213.24 ± 5.66^b^	231.39 ± 6.11^a^	0.0292
LYM (× 10^9^ L^−1^)	186.47 ± 5.32^d^	192.43 ± 5.32^c^	192.32 ± 4.23^c^	202.58 ± 4.22^b^	219.83 ± 3.83^a^	0.0475
MID (× 10^9^ L^−1^)	9.13 ± 0.45	9.42 ± 0.39	9.41 ± 0.67	9.92 ± 0.38	10.76 ± 0.56	0.4985
GRAN (× 10^9^ L^−1^)	0.69 ± 0.02	0.71 ± 0.02	0.71 ± 0.01	0.75 ± 0.05	0.81 ± 0.01	0.4993

Means followed by different small letters in the same row are significantly different (*P* < 0.05, one-way ANOVA). Abbreviations: *Hb*, hemoglobin; *Hct*, hematocrit; *RBCs*, red blood cell count; *MCV*, mean corpuscular volume; *MCH*, mean corpuscular hemoglobin; *MCHC*, mean corpuscular hemoglobin concentration; *WBCs*, white blood cells (as a total count); *LYM*, lymphocyte absolute count.; *MID*, mid-range absolute count; *GRAN*, granulocyte absolute count

### Plasma Biochemical Parameters

Plasma biochemical parameters are cleared in Table 5. Plasma ALT, AST, TC, TG, LDL-C, and VLDL-C levels noticeably reduced (*P* ≤ 0.05) by dietary supplementation of Fe, where the lowest significant values were in plasma of fish fed nano-Fe_2_O_3_. Furthermore, plasma albumin, total protein, and HDL-C demonstrated a considerable increase (*P* ≤ 0.05) in fish offered diet supplemented with Fe, where the highest values measured in fish received nano-Fe_2_O_3_.

**Table 5:** Plasma biochemical parameters and lipids profiles of Nile tilapia as affected by dietary additives of bulk and nano-Fe_2_O_3_

	Experimental treatments					*P* value
	Control	Bulk-Fe_2_O_3_ (mg kg^−1^ diet)		Nano-Fe_2_O_3_ (mg kg^−1^ diet)		
		0.2	0.4	0.2	0.4	
ALT (U L^−1^)	34.64 ± 0.85^a^	29.47 ± 0.69^b^	25.36 ± 0.87^c^	22.75 ± 0.92^ cd^	20.22 ± 0.96^d^	0.0001
AST (U L^−1^)	93.46 ± 1.25^a^	76.59 ± 1.54^b^	73.42 ± 1.33^bc^	68.67 ± 1.36^ cd^	64.81 ± 1.12^d^	0.0001
TP (g dL^−1^)	2.63 ± 0.18^b^	2.86 ± 0.19^ab^	2.97 ± 0.14^ab^	3.17 ± 0.19^ab^	3.34 ± 0.29^a^	0.0531
Al (g dL^−1^)	0.91 ± 0.01^c^	0.95 ± 0.02^c^	1.19 ± 0.02^b^	1.38 ± 0.01^a^	1.42 ± 0.03^a^	0.0001
Gl (g dL^−1^)	1.72 ± 0.18	1.91 ± 0.19	1.78 ± 0.14	1.79 ± 0.13	1.92 ± 0.21	0.9143
Al/Gl ratio	0.53 ± 0.07^ab^	0.50 ± 0.02^b^	0.71 ± 0.02^ab^	0.77 ± 0.01^a^	0.75 ± 0.03^ab^	0.0882
TC (mg dL^−1^)	221.48 ± 5.23^a^	200.64 ± 2.35^b^	181.34 ± 2.34^c^	162.55 ± 2.88^d^	135.67 ± 2.66^e^	0.0001
TG (mg dL^−1^)	130.55 ± 2.15^d^	135.67 ± 1.98^a^	181.24 ± 3.23^b^	162.52 ± 2.56^c^	135.78 ± 3.25^d^	0.0001
HDL-C (mg dL^−1^)	45.81 ± 0.95^e^	59.79 ± 1.11^d^	67.57 ± 0.89^c^	70.64 ± 1.12^b^	77.86 ± 1.11^a^	0.0001
LDL-C (mg dL^−1^)	149.56 ± 2.36^a^	100.74 ± 2.55^b^	77.52 ± 2.17^c^	59.40 ± 2.19^d^	30.65 ± 0.98^e^	0.0001
VLDL-C (mg dL^−1^)	26.11 ± 0.56^d^	40.11 ± 0.98^a^	36.25 ± 0.88^b^	32.50 ± 0.78^c^	27.16 ± 0.87^d^	0.0001

Means followed by different small letters in the same row are significantly different (*P* < 0.05, one-way ANOVA). Abbreviations: *ALT*, alanine aminotransferase; *AST*, aspartate aminotransferase; *TP*, total protein; *Al*, albumin; *Gl*, globulin; *Al/Gl ratio*, albumin/globulin ratio; *TC*, total cholesterol; *TG*, triglycerides; *HDL-C*, high-density lipoprotein cholesterol; *LDL-C*, low-density lipoprotein cholesterol; *VLDL-C*, very low-density lipoprotein cholesterol

### Plasma Lysozyme Activity and Antioxidant Biomarkers

**Fig. 1:**
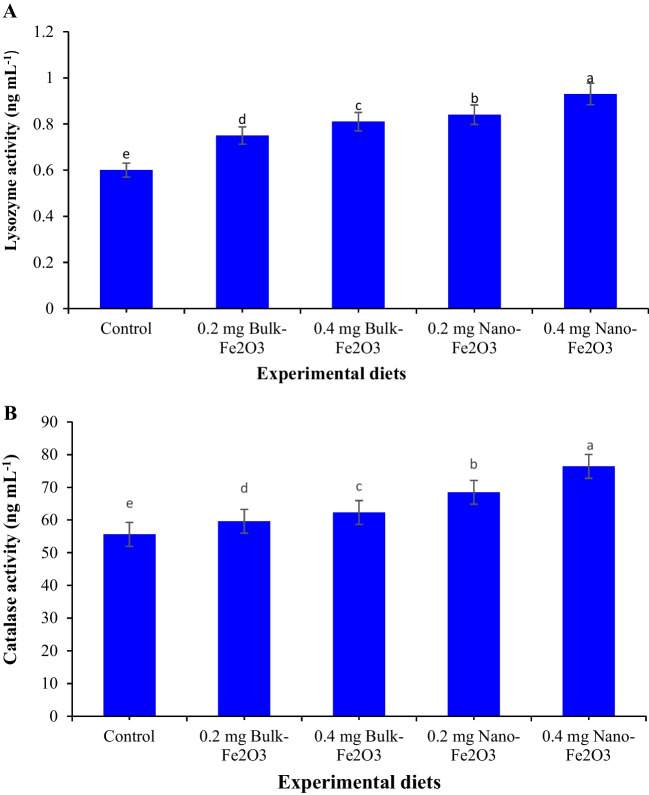
**A** Lysozyme activity, **B** catalase activity of Nile tilapia as affected by dietary additives of bulk and nano-Fe_2_O_3_. Different letters in columns indicate significant differences among treatments (*P* < 0.05)

**Fig. 2:**
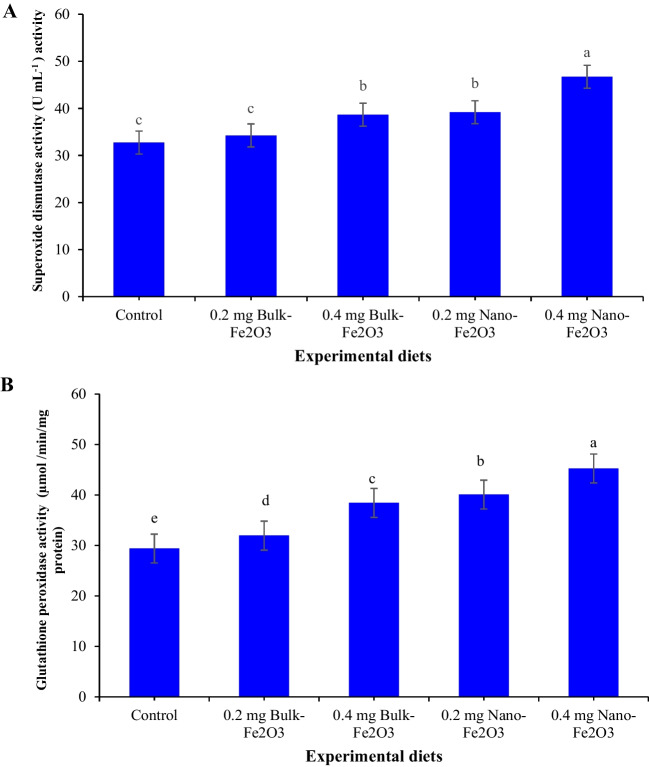
**a** Superoxide dismutase activity, **b** glutathione perioxidase activity of Nile tilapia as affected by dietary additives of bulk and nano-Fe_2_O_3_. Different letters in columns indicate significant differences among treatments (*P* < 0.05)

**Fig. 3:**
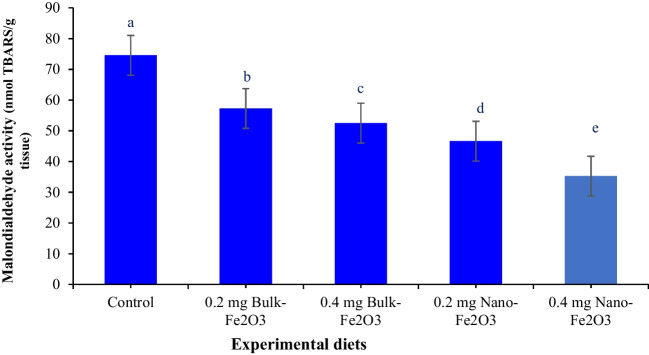
Malondialdehyde activity of Nile tilapia as affected by dietary additives of bulk and nano-Fe_2_O_3_. Different letters in columns indicate significant differences among treatments (*P* < 0.05)

Plasma lysozyme activity and antioxidant indicators are displayed in Figs. 1, 2, and 3. Plasma lysozyme activity, catalase (CAT), glutathione peroxidase (GPx), and superoxide dismutase (SOD) obviously rose (Fig. 1A and B and Fig. 2A and B) (*P* ≤ 0.05) in fish by dietary Fe inclusion, whereas the superior values reported in fish received 0.4 mg/kg^−1^ nano-Fe_2_O_3_ versus basal diet. The lowest value of MDA (*P* ≤ 0.05, Fig. 3) value was recorded in fish fed 0.4 mg/kg^−1^ nano-Fe_2_O_3_ versus other diets.

### Fish Fillet Nutrient Composition

Fish received nano-Fe_2_O_3_ included diets revealed a decline (Table 6, *P* ≤ 0.05) in the moisture content at both level of inclusion. While fat fillet value reduced as dietary adding level of Fe increased either in bulk or nano source, whereas the lowest fillet fat value observed in fish received 0.4 nano-Fe_2_O_3_ mg kg^−1^ diet. In contrast, crude protein and ash levels increased in fillet of fish fed on diet supplied with Fe compared to the control diet, the highest significant (*P* ≤ 0.05) levels observed in fillet of fish offered the nano-Fe_2_O_3_ provided diets. The best value of fish fillet Fe contents considerably increased (*P* ≤ 0.05) in fish fed diet supplemented with 0.4 nano-Fe_2_O_3_.

**Table 6:** Proximate analysis of flesh of Nile tilapia as affected by dietary additives of bulk and nano-Fe_2_O_3_

	Experimental treatments					*P* value
	Control	Bulk-Fe_2_O_3_ (mg kg^−1^ diet)		Nano-Fe_2_O_3_ (mg kg^−1^ diet)		
		0.2	0.4	0.2	0.4	
Moisture (%)	74.76 ± 0.88^a^	74.03 ± 1.12^b^	73.17 ± 1.47^c^	72.74 ± 2.13^d^	72.49 ± 2.11^d^	0.0001
Protein (%)	18.84 ± 0.14^d^	20.14 ± 0.19^c^	20.72 ± 0.56^b^	21.66 ± 0.38^a^	21.96 ± 0.78^a^	0.0001
Fat (%)	2.93 ± 0.12^a^	2.13 ± 0.21^b^	1.94 ± 0.13^bc^	1.62 ± 0.14^bc^	1.47 ± 0.15^c^	0.0022
Ash (%)	2.04 ± 0.13^d^	2.52 ± 0.90^c^	2.75 ± 0.56^b^	3.08 ± 0.02^a^	3.20 ± 0.02^a^	0.0001
Fe_2_O_3_ (mg kg^−1^)	14.14 ± 0.04^d^	16.22 ± 0.02^d^	19.85 ± 0.06^c^	19.29 ± 0.06^b^	21.18 ± 0.07^a^	0.0001

Means followed by different small letters in the same row are significantly different (*P* < 0.05, one-way ANOVA). Abbreviations: *Fe*_*2*_*O*_*3*_, iron oxide

## Discussion

Growth and feed utilization markedly increased in tilapia received diet augmented with Fe versus to those fed the basal diet. The present study showed the best performance observed in fish groups fed on 0.4 Nano-Fe_2_O_3_ mg kg^−1^ diet. Besides, tilapia survival rate augmented significantly by inclusion of dietary addition of Fe either from bulk or nano source. The results agreed with Khan et al. [35] who observed that nano-nutrients complex (Zn, Se, Fe, and Cu) mixed with basal diet displayed the best growth traits; 33% higher final weight, better SGR and FCR values than feeding with a commercial basal diet. Similarly, [36] observed the elaboration effect of the diets containing iron nanoparticle on trout growth compared with the inorganic form. Such positive outcomes of nano iron diet recorded in tilapia [37].

Moreover, it has confirmed by [8] who found that basal diet provided with FeNPs improves the final weight of treated *Labeo rohita*. Similarly, improvement of growth and survival has been noticed in different fish species fed SeNP and ZnNP supplied diet [38, 39]. The present results could be attributed to the following reasons: (i) FeNPs ease nutrient assimilation that accordingly progress growth and feed efficiency [40], (ii) the introduction of FeNPs upsurge the specific surface area of compounds [41], (iii) higher bioavailability and utilization efficacy of of FeNPs compared to other forms of selenium [14, 42], (iv) the function of FeNPs as the nutrients trailer (especially AAs), these nutrients could pass into blood as a source of energy [41], (v) FeNPs construct as enzyme-cofactor indorse the breakdown and absorption of nutrients, (vii) FeNPs act as stimulatory and regulatory factor of bone formation and mineralization [43], (viii) FeNPs rapidly taken up by the cells, and exhibit higher bioavailability which accordingly enhance growth [42, 44], and (ix) the biological role of Fe as immunostimulant on bone formation, mineralization, and hematological parameters of fish [45, 46].

The number of goblet cells, villus width/length, MSR, and absorption area are important markers of intestinal morphology because they play a substantial part in nutritional absorption by extending and changing the absorption area of the fish intestine, resulting in better performance [47]. The intestinal topography as absorption area of villous (AAV) of anterior intestine noticeably increased in fish fed 0.2 mg kg^−1^ nano-Fe_2_O_3_, and in posterior intestine AAV and mucosal to serosal amplification ratio (MSR) values improved obviously in fish fed nano-Fe_2_O_3_ at both levels 0.2 and 0.4 mg kg^−1^. MSR of anterior intestine significantly rose in fish group received 0.4 mg kg.^−1^ nano-Fe_2_O_3_. The results indicate normal growth, health, and integrity of the intestinal structure. The amelioration of intestinal structure of tilapia due to iron supplementation particularly the nano form, which displayed the best measures, indicates that Fe is an essential trace element, and its dietary addition is required to meet the fish nutritional need and to perform its physiological function in regulating the normal tissues growth, immune, and the antioxidative defense and can protect from cell damage [8, 45, 46]

Haematological indices are displayed the physiological, immunity, and health status of fish, as well as disease and metabolic disorders [48–51]. Also, leukocyte differentials act as the first line of defence against external invasions into the organism’s system [52]. The hematological parameters herein including: hemoglobin, hematocrit concentration, WBCs, and LYM cell count were the best in fish received nano-Fe_2_O_3_ mg kg^−1^. It confirms the biological role of Fe in the blood functions. The same amelioration effect of nano nutrients on the hematological parameters has been revealed by [37] who found the most haematological parameters, such as RBCs, hematocrit, and hemoglobin, were higher in trout fed nanoparticulate treatments than in the inorganic ones. Furthermore, [8] reported a substantial rise in the hematological parameters such as RBCs, hemoglobin of rohu fed the nano-Fe-fortified diets compared with the inorganic iron and the iron-deficient diet. The present results could be attributed to the following: (i) high availability of nano-Fe_2_O_3_ uptake on nanoparticle form. (ii) Iron is a chief constituent of RBCs [53]. Red cell indices (MCV, MCH, and MCHC) are key biomarkers that give helpful information on haemoglobin concentration and red blood cell sizes [49], and are therefore utilized to diagnose anaemia in animals (Yaji et al., 2018b). Changes (elevation and/or decrease) in red cell indices outside the normal physiological range usually indicate microcytic or macrocytic anaemia [54]. In the current data, there were insignificant differences in diets supplemented with either bulk-Fe or nano-Fe indicating normal physiological status of tilapia.

Alanine aminotransferase (ALT) and aspartate aminotransferase (AST) enzymes in the serum of the blood are involved in cellular nitrogen metabolism, oxidation of amino acids, hepatic gluconeogenesis, and hepatic status, and their high levels in fish plasma could cause liver dysfunction [33, 55]. The lipid profile, which includes cholesterol and triglycerides, can change depending on nutritional status [56]. Triglycerides (TG) are measured to monitor lipid metabolism. High TG levels can cause glycogen storage disease, nephritic syndrome, and liver failure [57, 58]. The present study showed that fish fed nano-Fe_2_O_3_ recorded the lowest (*P* ≤ 0.05) values of ALT, AST, TC, TG, LDL-C, and VLDL-C levels compared to those fed control and bulk-Fe_2_O_3_. The current findings are in parallel with [37] who found positive effect of rainbow trout fed diets supplemented with nanoparticulate iron and copper on liver enzymes, blood biochemical parameters, antioxidant response, and immune function.

Furthermore, [38] noticed that inclusion of nano-Fe reduced the AST and ALT activities in Nile tilapia fish. The present results could be explained by the following: (i) the role of FeNPs in improving the fish health [46] and (ii) the role of FeNPs of decreasing the toxic and the stressful factors such as liver cell degeneration and necrosis which could increase the levels of ALT and AST [38, 46]. The present findings showed an improvement of plasma albumin, total protein, and HDL-C in fish offered diet supplemented with Fe, where the uppermost values measured in fish received nano-Fe_2_O_3_. Such effect has been noticed by [37] who detected those levels of serum total protein, albumin, and globulin contents in trout improved with Fe supplementation, where the highest values determined in fish fed with the nanoparticulate diets versus inorganic and control ones. Thus, confirming that minerals have beneficial in controlling the blood-protein synthesis in animals, and diets provided with minerals enhance the protein synthesis in fishes [7, 59, 60]. Also, it confirms that nano iron supplied diets were more potent in stimulation of blood protein synthesis. The increase of total protein level is linked with efficient protein utilization, increase in growth, and strong immunity due to the level of total protein gives evidence about the health, nutritional, and immune status [61, 62]. Also, albumin level improved in fish plasma fed nano iron indicating the higher ability of hepatocytes to synthesis the albumin and the efficient protein utilization. Likewise, [38] in tilapia and [62] in *Clarias batrachus* stated the effect of nano iron supplementation in increasing of blood proteins concentrations.

The lysozyme activity has a link with leucocytes and is produced mostly by macrophages, is the most important sign of the immune response as a result of many immune stimulants and microbiological components [63, 64]. The antioxidant enzymes revealed the body’s antioxidant system’s functional status, which reflected the body’s ability to break down oxygen-free radicals and to protect the tissues of the fish from oxidative damage. However, there is a link between antioxidant defense and fish responsiveness in aquaculture [65]. SOD is an antioxidant enzyme that catalyses the dismutation of superoxide anion into hydrogen peroxide and is therefore the first enzyme linked with the antioxidant defence line [66]. The GPx enzyme protects the animal against oxidative damage by converting hydroperoxides to alcohols [67]. Catalase activity plays a protective role against external superoxide sources by assuring the elimination of free hydrogen perioxide radicals. Lysozyme, CAT, GPX, and SOD obviously rose herein in fish by dietary Fe supplementation, whereas the uppermost values reported in fish received 0.4 mg kg^−1^ nano-Fe_2_O_3_ compared to fish fed other diets. The lowest value of MDA activity was recorded in fish fed 0.4 mgkg^−1^ nano-Fe_2_O_3_. The present results are in parallel with [37] who demonstrated that in general the addition of Fe improved the antioxidant enzymes and lysozyme activity, especially in the nanoscale form which revealed the highest values, compared with the bulk selenium and basal diets. The findings could be attributed to the following theories: (i) FeNPs decreasing the stressful conditions and promoting the innate immune parameters [46] and (ii) the present result highlight the importance of Nano technologies as an antioxidant component and quicken electron transfer. The present result of MDA assures the improved antioxidant defense of tilapia fed Fe supplied diets particularly in the nanoparticle form. Therefore, nano iron could be added to farm animal feed to avoid deteriorative oxidative processes or to promote oxidative stability in order to maintain food quality and nutritional value.

Fillet of fish received nano-Fe_2_O_3_ diets revealed an obvious decline in the moisture content at both level of inclusion compared to those fed the control and bulk-Fe_2_O_3_ diets. While values of fat in fillet reduced with increasing the inclusion levels of Fe either in bulk or nano source, whereas the lowest fillet fat value observed in fish received 0.4 nano-Fe_2_O_3_ mg kg^−1^ diet. The present results are in parallel with [36] who found that the fat and trans-fat contents of the fish fed diets contained nano-Fe_2_O_3_ were lower than those fed the basal diet. While the protein and ash levels increased in fillet of fish fed on diet supplied with Fe compared to the control diet, the highest significant values observed in fillet of fish fed nano-Fe_2_O_3_ diets. The current findings are in parallel with [36] who found that protein content in muscle of fish fed nano-nutrients higher than other groups. Fe content considerably increased in the fillet by dietary supplementation of Fe, whereas the best values recorded in fillet of fish fed nano-Fe_2_O_3_ mg kg^−1^. The present results are in parallel with [36] who found that high nutrients content of Fe in the muscles of fish fed with nano nutrients versus to those treated with a commercial diet. The current findings could be attributed to the following: (i) higher bioavailability of nano nutrients forms and (ii) nano-Fe_2_O_3_ has better connections with other substances and could be absorbed, retained, and reserved simply and more competently in the fish body [42–44].

In conclusion, Fe is required to satisfy the nutritional needs of the fish as well as perform its physiological function in regulating proper tissue development, immunological and antioxidative defense, and cell damage protection. Thus, adding Fe in nano form to tilapia diets improves performance, immunological, fillet composition, and the healthiness of the intestinal structure more effectively than control-free Fe diets and bulk-Fe_2_O_3_ provided diets. However, further investigations for expression of some genes related to growth and antioxidant capacity are needed to clarify the function of nano-Fe_2_O_3_ on growth and health statues of fish.

## Data Availability

The datasets generated during and/or analyzed during the current study are available from the corresponding author on reasonable request.
